# Supplementary material for: Differences in the Clinical and Molecular Profiles of Subungual Melanoma and Acral Melanoma in Asian Patients
Source: Cancers (Basel). 2023 Sep 4;15(17):4417. doi: 10.3390/cancers15174417 (PMC10486359; doi:10.3390/cancers15174417)
Supplement: Supplementary file 1 [file cancers-15-04417-s001.zip › cancers-2568908-supplementary.pdf]

Supplementary Materials

Differences in the Clinical and Molecular Profiles of Subungual Melanoma and Acral Melanoma in Asian Patients

So-Young Ahn, Go Eun Bae, Seung-Yeol Park and Min-Kyung Yeo

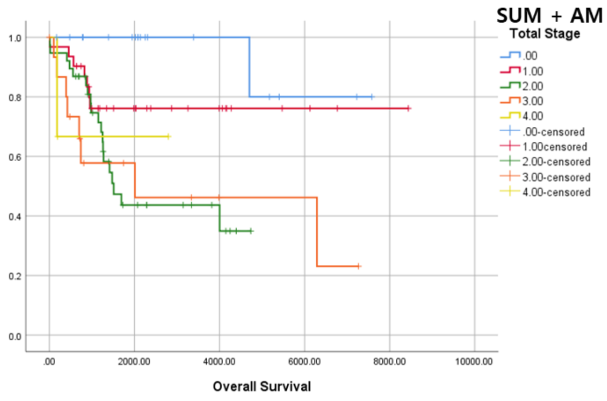

Log Rank (Mantel-Cox)	15.477	4	.004
Breslow (Generalized Wilcoxon)	13.804	4	.008

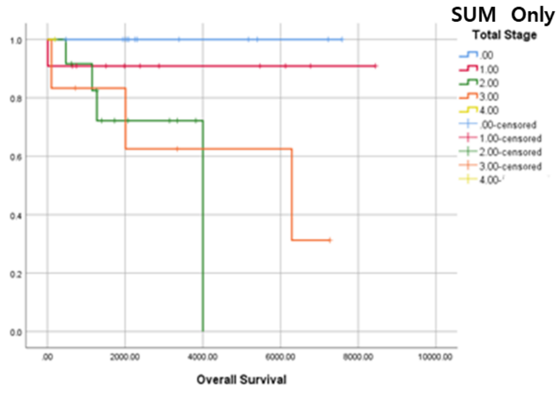

Log Rank (Mantel-Cox)	8.473	4	.076
Breslow (Generalized Wilcoxon)	5.169	4	.270

Figure S1. Kaplan–Meier curves showing overall survival of patients with SUM and AM stratified according to the stages. (left) All melanoma patients with SUM and AM, including in situ melanoma; (right) patients with invasive melanoma.

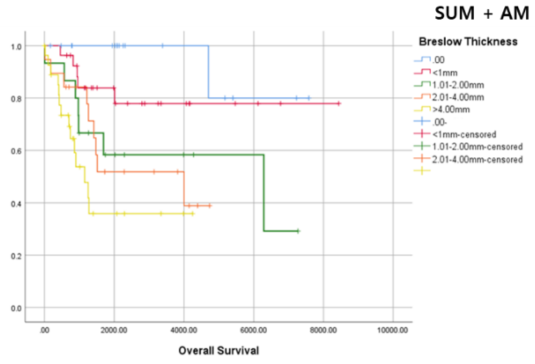

Log Rank (Mantel-Cox)	22.077	4	.000
Breslow (Generalized Wilcoxon)	20.963	4	.000

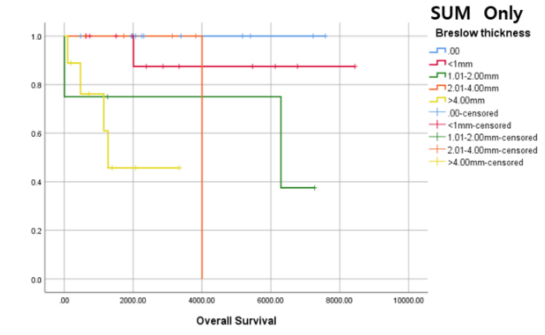

Log Rank (Mantel-Cox)	14.568	4	.006
Breslow (Generalized Wilcoxon)	13.239	4	.010

Figure S2. Kaplan–Meier curves showing overall survival of patients with SUM and AM stratified according to the Breslow thickness. (left) All melanoma patients with SUM and AM, including in situ melanoma; (right) patients with invasive melanoma.

Table S1. Demographic data of 42 patients with subgual melanoma during 15 years.

Characteristics	Patients Number (%)
Age at diagnosis (mean)	55.7
**Sex**	
Male	20 (47.6)
Female	22 (52.4)
**Tumor Location**	
Upper	29 (69.0)
Lower	13 (31.0)
Size (mean, cm)	1.2 cm
**Breslow Thickness**	
In situ	12 (28.6)
<1 mm	12 (28.6)
1.01-2.00mm	4 (9.5)
2.01-4.00 mm	5 (11.9)
>4.00 mm	9 (21.4)
**Clark Level**	
1	12 (28.6)
2	11 (26.2)
3	6 (14.3)
4	5 (11.9)
5	8 (19.0)
**Ulceration**	
Absent	31 (7.38)
Present	11 (26.2)
Mitotic rate/mm^2 (mean)	3.0/mm^2
**Lymph Node Metastasis at the Time of Diagnosis**	
Absent	35 (83.3)
Present	7 (16.7)
**Distant Metastasis at the Time of Diagnosis**	
Absent	41 (97.6)
Present	1 (2.4)
**Stage Group**	
0	12 (28.6)
I	11 (26.2)
II	12 (28.6)
III	6 (14.3)
IV	1 (2.4)
**Chemotherapy**	
Not done	30 (71.4)
Done	12 (28.6)
**Radiotherapy**	
Not done	39 (73.8)
Done	3 (26.2)
**Immunotherapy**	
Not done	31 (73.8)
Done	11 (26.2)
**Trauma**	
Absent	37 (88.1)
Present	5 (11.9)

Table S2. Gene lists for Oncomine Comprehensive Assay version 3 (n=170).

Small nucleotide variant and indels (Hotspot genes, n=170, from DNA)

ABL1, ABL2, AKT1, AKT2, AKT3, ALK, APC, AR, ARAF, ASXL1, ATM, ATR, AURKA, AURKB, AURKC, AXL, BAP1, BCL2, BRAF, BRCA1, BRCA2, BRD2, BRD3, BRD4, CBFB, CCND1, CCND2, CCND3, CCNE1, CDH1, CDK12, CDK4, CDK6, CDKN1A, CDKN1B, CDKN2A, CDKN2B, CDKN2C, CEBPA, CHEK2, CREBBP, CRKL, CSF1R, CTNNB1, DDR1, DDR2, MAP3K4, MAPK1, MAPK3, MAPK8, MCL1, MDM2, MDM4, MED12, MEN1, MET, MITF, MLH1, MPL, MSH2, MSH6, MTOR, MYC, MYCN, MYD88, NF1, NF2, NFKBIA, NKX2-1, NOTCH1, NOTCH2, NOTCH3, NOTCH4, NPM1, NRAS, NTRK1, NTRK2, NTRK3, NUTM1, PDGFB, PDGFRA, PDGFRB, PIK3CA, PIK3CB, PIK3CD, PIK3R1, PIK3R2, POLE, PPARG, PTCH1, PTEN, RAB35, RAD50, RAF1, RARA, RB1, RET, RHEB, RICTOR, RNF43, ROS1, RSPO1, RSPO2, RUNX1, SMAD2, SMAD4, SMARCA4, SMARCB1, SMO, SRC, STK11, SYK, TET2, TMPRSS2, TOP2A, TP53, TSC1, TSC2, VHL, WT1, XPO1, ZNRF3, TERT

Copy number variation (n=47, from DNA)

AKT1	FGFR1	MDM2	PPARG	CCND3	NTRK2
AR	FGFR2	MDM4	TERT	CDK2	NTRK3
CCND1	FGFR3	MET	AKT2	CDKN2A	PDGFRB
CCNE1	FGFR4	MYC	AKT3	CDKN2B	PIK3CB
CDK4	FLT3	MYCL	ALK	ESR1	RICTOR
CDK6	IGF1R	MYCN	AXL	FGF19	TSC1
EGFR	KIT	PDGFRA	BRAF	FGF3	TSC2
ERBB2	KRAS	PIK3CA	CCND2	NTRK1	

Fusions and splice variants (n=51 from RNA)

ALK	FGFR1	ROS1	FLT3	NOTCH4	RAD51B
AXL	FGFR2	AKT2	JAK2	NRG1	RB1
BRAF	FGFR3	AR	KRAS	NTRK2	RELA
EGFR	NTRK1	BRCA1	MDM4	NUTM1	RSPO2
ERBB2	NTRK3	BRCA2	MET	PDGFRB	RSPO3
ERG	PDGFRA	CDKN2A	MYB	PIK3CA	TERT
ETV1	PPARG	ERBB4	MYBL1	PRKACA	
ETV4	RAF1	ESR1	NF1	PRKACB	
ETV5	RET	FGR	NOTCH1	PTEN	

Table S3. Melanoma patients enrolled in pathology archives in a single institute (CNUH) from 200 to 2022 (15 years).

**Cutaneous**			245	81%
	Acral	151		50%
	Subungual (28% of AM)	43		14%
	Other Cutaneous (Superficial Spreading, Nodular, Lentigo Maligna)	94		
**Mucosal**			47	16%
	Oral and Nasal	35		
	Digestive Tract (Including Anorectal)	7		
	Vulvovaginal	4		
	Genitourinary (Including Penis)	1		
**Ocular**	Including Conjunctiva		10	3%
**Total**			302	100%
